# Investigating the Effects of Fiber Reinforced Concrete on the Performance of End-Zone of Pre-Stressed Beams

**DOI:** 10.3390/ma12132093

**Published:** 2019-06-28

**Authors:** Asif Jalal, Nasir Shafiq, Muhammad Zahid

**Affiliations:** Civil Engineering, Universiti Teknologi PETRONAS, Seri Iskandar 32610, Malaysia

**Keywords:** end zone, fiber reinforced concrete, PVA fibers, post-tensioning

## Abstract

This paper presents the results of the behavior of end zone of post-tensioned (PT) beams made of fiber reinforced concrete (FRC). The principal aim of using FRC was to enhance the ductility and post-cracking behavior of end-zone of post-tensioned beams. A stronger and tougher end-zone of PT-beams is necessary when it is subjected to dynamic loading. Post-tensioned (PT) beams are typically used for the construction of bridges and industrial buildings, which are often subjected to vibrations and cyclic loading. Pre-mature cracking of the end zone (EZ) of a PT-beam is considered the type of problem that may cause the structural collapse. In this research program, polyvinyl alcohol (PVA) and copper-coated steel (CCS) fibers were used in concrete for improving the EZ performance of PT-beams. The use of FRC caused a 50% reduction in the shear reinforcement within the end zone of the PT-beam, which also avoided the congestion of steel in the end zone. Hence, the concrete was placed homogeneously, and smooth finished surfaces of the beams were obtained. FRC controlled the bursting of the end zone during the transfer of the full pre-stress force, and approximately 25% increment in the strain energy of the end zone was observed, which was also found efficient in strain diminution along the length of the beam.

## 1. Introduction

For the past several years, the advances made in the field of prestressed-concrete have widened the freedom of architectural setting of floor-framing in a building structure. Architectural freedom allows achieving high loading capacity with the adoption of longer-span and smaller cross-section dimensions. Post-tensioning technique of prestressing is the most commonly adopted method by the industry due to many advantages. The post-tensioned bar in a concrete member constitutes concentrated force applied under compression mode to the concrete cross-section. It has been experienced that the application of high magnitude focused force to the immature concrete section is always a big challenge. In the early development stage from the 1950s to 1980s, many infrastructures made of pre-stressed girders suffered pre-mature failures. For example, Stone & Breen [1] reported the observation of the appearance of cracks at an early age in many bridges in the state of Florida. Similar comments are made in California and Texas [2] and during the construction of stadiums for Montreal Olympic in the thin slabs and webs structure [3]. Upon investigation, it was found that the cracks were formed due to the transmission of high intensity of compressive and tensile forces in the anchorage zone of the pre-stressed concrete beam. The formation of such types of cracks may result in the loss of prestressing strength, which sometimes leads to in-service failure.

In many cases, to avoid such types of failure; heavy shear reinforcement is usually introduced within the end anchorage zone to stop the crack propagation. It is observed that the cracks form a passage within the concrete that allows aggressive agents present in the vicinity of the structure to ingress in the concrete that can cause corrosion to the embedded steel reinforcement [4]. Once rusting of embedded steel bars initiated, it continues to progress, and upon reaching the threshold level called the critical corrosion of embedded steel bars, might lead to spalling of concrete and eventually results in a structural failure.

Breen et al. [5] have classified the anchorage zone as two types; local and general zones on the bases of stress distribution. By applying the high amount of prestressing force to the concrete section inducing the high magnitude of bearing stresses and tensile bursting stresses along with the profile of the prestressing tendon. Tensile stresses develop along the edges of the lateral loading called the spalling-stress, whereas, the tensile stress evolves at the longitudinal side is known as the longitudinal edge tensile stress [6,7].

There are several factors responsible for the unusual behavior of anchorage zone; the most dominating factors are section dimension, eccentricity, loading area, external influence, reinforcement, anchorage device, inclination, and the number of tendons and properties of concrete. Cracks develop along with the longitudinal edge to form a link between inside reinforcement and the ambient environmental conditions. The mode of longitudinal edge tensile stresses are changed to compressive stress at a farther distance from the end [6], but this phenomenon may change when there is less or no skin reinforcement. The area of compression and tensile portions and peak tensile value depends on anchorage end conditions. The increase in the eccentricity and loading area (bearing plate size) decreases the bursting stresses, shift the peak bursting force point away from the loading face, and amplify the edge spalling stresses [8]. The experimental study showed that the addition of transverse reinforcement enhances the end zone ductility and, hence, the load-carrying capacity [9]. Some researchers proposed considering bending tensile stresses when large eccentricity occurred, which acts farther than the anchoring point [7]. Therefore, identification of the local and general zone is crucial and require special consideration during the design phase, especially in the case of the high loading area and large eccentricity.

In earlier studies, high strength concrete (HSC) was introduced in dealing with the issues and challenges faced regarding the performance of end anchorage zone of post-tensioned beams, the aim of using HSC was to resist the high compression stresses. Whereas, additional shear reinforcement was added to the conventional shear reinforcement to withstand the tensile stresses (AASHTO, 2002). Addition of low elastic modulus fiber reinforced polymer tendon reduces losses and increases prestressing level [10]. Some researchers proposed extra supplementary reinforcement such as orthogonal reinforcement, grid type mesh [11] and spiral reinforcement, which can further enhance the performance of end anchorage zone [1]. Usually, HSC contains a high amount of cement that increases the brittleness of concrete, whereas the additional reinforcement makes the end zone congested, hence results in the poor consolidation of concrete [12]. Because of the new problems arising due to the brittleness of HSC, the concept of enhancing the ductility of concrete by adding the appropriate type of fibers has been appreciated by researchers that can eliminate the need for additional reinforcement, which can avoid the congestion of reinforcement. In a research study, Tawfiq and Robinson [13] reported that the addition of 0.5% steel fibers in concrete caused a reduction of 40% mild steel reinforcement content, whereas in another research investigation, adding of 0.75% hook-shaped steel fibers in concrete resulted in 100% elimination of the skin reinforcement [14]. Yazdani et al. [15] used 1% of steel fibers by volume fraction in concrete that replaced 100% skin and spiral reinforcement content. Although many successes discussed as above with the use of steel fiber reinforced concrete, however, it also faced various issues and criticism; Granju and Balouch [16] noticed corrosion of fibers when exposed to the severe condition, whereas Chanh [17] experienced some difficulties during mixing of concrete. In recent studies, some efforts are made to overcome the issues faced by using steel fibers in concrete. For example, copper coated steel fibers are introduced to avoid corrosion problems. Using copper coated micro steel fibers, Sun and Wan [18] successfully enhanced the ductility of cementitious composites. In another research investigation, Ranjbar et al. [19] introduced copper coated steel (CCS) fibers in the geopolymer matrix. Some researchers proposed to replace steel fibers with non-metallic fibers as an alternative form of FRC. In a recently conducted experimental program, polyvinyl alcohol (PVA) fibers were introduced in fiber reinforced concrete (FRC). The addition of PVA fibers caused a significant improvement in the performance of concrete; a worthwhile increase was observed in the compression, flexure, and tensile strength of concrete [20,21]. Some research studies highlighted that PVA fibers remarkably improved the post-cracking behavior and ductility of concrete [22,23]. Many researchers observed that PVA fibers in concrete perform better than steel fibers. After noting the significant effects of PVA fibers in improving the performance of concrete, it was hypothesized that PVA fiber reinforced concrete would be a better candidate for developing the high-performance end zone of the PT-beams. PVA fibers in concrete help in avoiding the congestion of conventional steel in the end-zone of PT-beams and enhance the post-cracking behavior. For the past several years, fly ash has become a popular ingredient for producing green and sustainable concrete [24]; it improves the workability [25,26] and long-term strength characteristics of plain concrete [27]. However, minimal research was found for the application of fly ash based concrete application in the PT-beams. Recently, it has been found that application of non-metallic fibers like PVA fiber and copper coated steel fiber is the best substitute for conventional steel fiber without any corrosion issue. The research on these fibers is still in progress and is therefore not yet applied to structural elements such as prestressed concrete. This research is pure novelty base study, which employs fly ash, PVA, and CCS fiber reinforced concrete research findings in a more refined manner to address the shortcomings of Post-tensioning technology.

The principal aim of this research study was to investigate the performance of the end zone (EZ) of PT-beams made of FRC; two type of fibers; copper-coated micro steel (CCS) and PVA fibers were used. The concrete mixes were developed using 100% cement as well as 80% cement + 20% fly ash. The first phase of the research program focused on investigating the effects of fiber content on the workability and mechanical properties of concrete, whereas the second phase of the research was to evaluate the contribution of PVA and CCS fibers in resolving performance concerns of the end-zone of PT beams. 

## 2. Research Significance

The results discussed in this experimental investigation are significant for improving the performance of the anchorage end zone of post-tensioned concrete beams, which usually face challenges of avoiding the premature cracks and failure. Therefore, the enhancement of the performance of the anchorage end zone by increasing the ductility of concrete using PVA or CCS fibers results in avoiding cracks at the early stage of the beam, which can improve the durability of the beam because of the resistance of ingress of the aggressive agent. 

### 2.1. Materials and Mix Proportion

Ordinary Portland cement (OPC) type-1 and fly ash (FA) Class-F conforming to ASTM C150 and ASTM C618-15 respectively were used in this study. Well-graded coarse aggregates (4.75 mm to 20 mm) and river sand were acquired from a local query in Perak, Malaysia. Superplasticizer Sika Viscocrete 2044 was used to improve the workability of fresh concrete. Two types of fibers, PVA fibers 18 mm length and 200 μm diameter (aspect ratio 90) and CCS fibers of 20 mm length and 200 μm diameter (aspect ratio 100), were bought from off the shelf. 

A total of six different mixes was prepared; three mixes were made of 100% OPC, and the other three were made of 20% FA + 80% OPC. 100% OPC mix with no fiber is called the control mix (CM), and the mix with 20%FA without fiber is called the control mix (CF). Table 1 shows the properties of fibers used in this study, and Table 2 shows the details of mix proportions. From an earlier study on fiber reinforced concrete, the mix proportion was selected as 1:1.66:2.59 with a water to binder ratio of 0.4 [28]. Fiber content was chosen as 0.3% by the volume fraction of the binder content. Mixes labeled P0.3 and S0.3 have a volume fraction of 0.3% PVA fiber and CCS fiber respectively, in the control mix having 100% OPC, while PF0.3 and SF0.3 identify the inclusion of fly ash (as 20% replacement of cement) in fiber reinforced concrete mixes. 

### 2.2. Test Specimens

**Figure 1:**
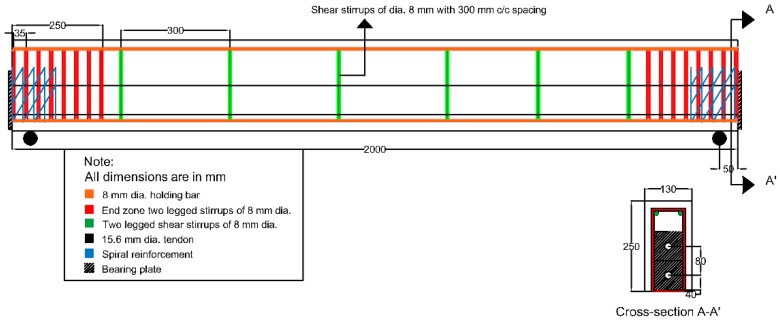
Detail diagram of the control beam (unit: mm).

All samples were cast and cured according to requirements of ASTM C192/C192M-16a. 100 mm size cubes were made for compressive strength test and 100 mm diameter and 300 mm high cylinders were made for split tensile and elastic modulus tests, whereas 100 mm × 100 mm size and 500 mm long prism were cast for the flexural strength test. Dimensions of PT-Beams were designed as 130 mm wide, 250 mm high, and 2000 mm long. Ducts for insertion of PT-Tendon was placed in the formwork before concreting for beam; two unbonded tendons comprising of seven wire strands passing through black anti-UV treated HDPE were used. Tendon nominal diameter was selected as 15.3 mm, and tensile strength was 1860 MPa, which complied with the requirements of EN 10138 and BS 5896. For improving the bursting and spalling stresses, one tendon was inserted at the center, and the other was placed at an eccentricity of 80 mm. Both tendons were coupled with a bearing plate and spiral reinforcement at the end. Eight shear links were placed within the anchorage block of normal concrete (CM & CF) beams while FRC beams contained only four shear links (50% reduction). Figure 1 shows the reinforcement and other details of the control PT beams. Zhang et al. [7] discussed the procedure of the selection of end zone parameters for analysis. Ratio of loading area to member cross-sectional area (k) is 0.4, ratio of eccentricity to member height (e/h) is 0.32, and λ is 0.64 (2e/h).

**Figure 2:**
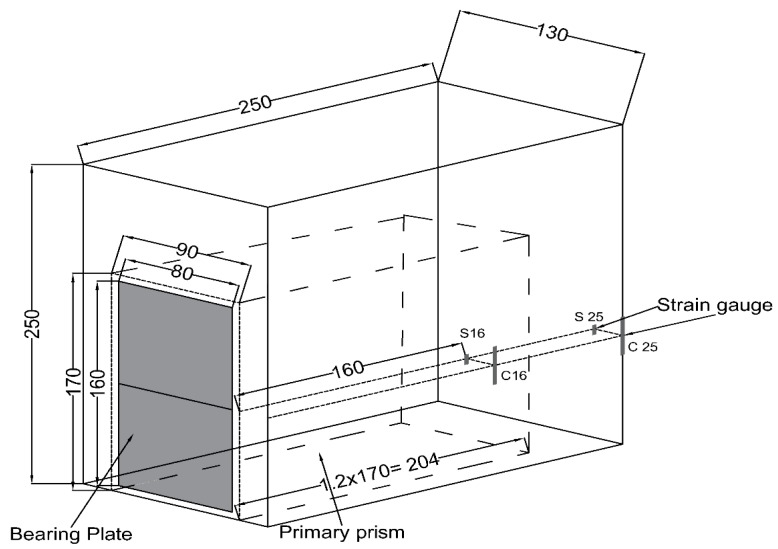
End zone of post-tension prestress beam (unit: mm).

Four strain gauges were installed within the end region for monitoring the performance of the anchorage zone for measuring the variation in the magnitude of the prestressing force. Two strain gauges were glued on the stirrups, and the other two were attached to the concrete surface at the distance of 160 mm and 250 mm from the end of the beam. Figure 2 illustrates the details of the anchorage block as described by the Eurocode-2 [29], and it also shows the location of the strain gauges. 

A total number of six beams were cast; three of them were made of fly ash concrete, and the other three were cast using 100% OPC concrete. When prestressing was applied, fly ash concrete beams suffered cracks before reaching the pre-decided value of the prestressing force of 200 kN. The reason could be the low early strength of fly ash concrete. The pozzolanic effects of fly ash appear after 28 days. It was obtained that at the early age fly ash concrete beams resist lower initial force than the 100% OPC concrete. Therefore, the fly ash based beams were prestressed until the hairline cracks appeared on the surface, Table 1 shows the details of each of the beams. All beams I.D begins with “PSC,” which indicates that the beam is a prestressed concrete beam, while the edition of “fy” indicates the 20% replacement of OPC by fly ash. “PVA-0.3” and “CCS-0.3” in the nomenclature specify the addition of 0.3% volume fraction of polyvinyl alcohol (PVA) fiber and copper coated steel (CCS) fiber respectively.

### 2.3. Testing and Post-Tensioning Methodology 

**Figure 3:**
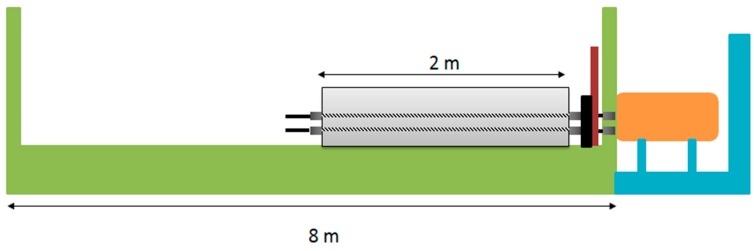
Schematic description of the post-tensioning bed installed in UTP.

Cube compressive strength test was done at the age of 3, 7, 28, and 56 days; the test was performed according to BS EN 12390-3:2009. Split-tensile, flexure, and elastic modulus tests were conducted at the age of 28 days only. Post-tensioning of beams was done at the 7th-day of concrete casting, using prestressing bed available in the concrete technology laboratory, Universiti Teknologi PETRONAS. The details are illustrated in Figure 3. The live end consisted of two steel plates attached with a thick circular plate to restrain the movement of the beam—the thick circular plate containing holes for tendons to pass through, which only allows the one-way flow of tendons with the help of wedge and barrel. However, wedges and barrel are attached at the dead end, which effectively restrained the inward movement. Details of PT are given in Table 3, whereas Table 4 shows the details of the entire experimental program designed for this study.

## 3. Results and Discussion

### 3.1. Effects of Fiber Addition on the Properties of Concrete

**Figure 4:**
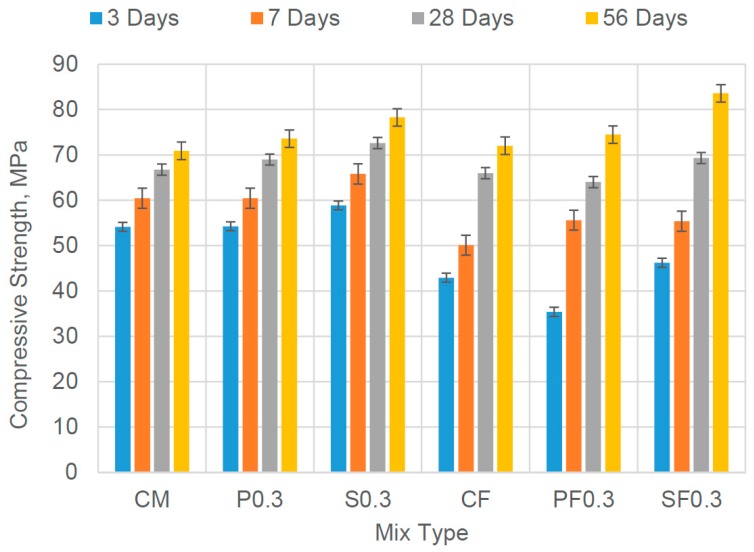
Compressive Strength Test Results of all Concrete Mixes.

Experimental test results of compressive strength of all concrete mix are given in Table 5, whereas Figure 4 shows the trend of development of compressive strength at different age of testing. The workability in terms of slump value of all concrete mixes was set 80 ± 10 mm; therefore, a dosage of superplasticizer (SP) was added if the desired slump was not achieved. The mix design was based on the 28 days’ target cube compressive strength of 60 MPa. All concrete mixes in this study qualified for the desired value of the compressive strength. It is noticed that the concrete mixes containing PVA and CCS fibers showed a little higher amount of compressive strength as compared to the respective control mix.

**Figure 5:**
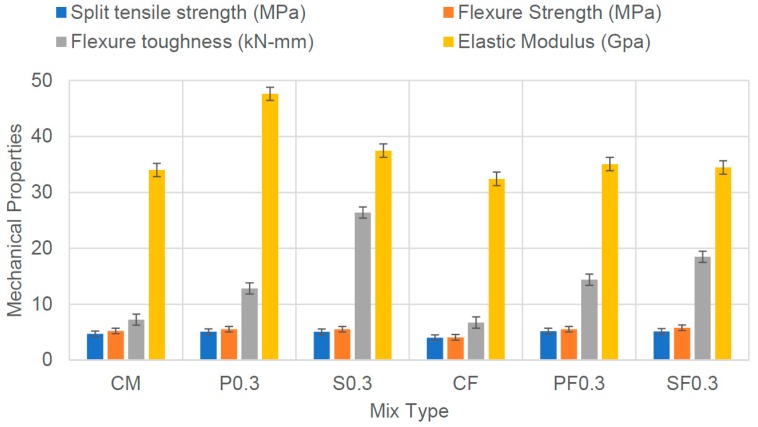
Mechanical Properties of all Concrete Mixes Measured at 28 Days.

**Figure 6:**
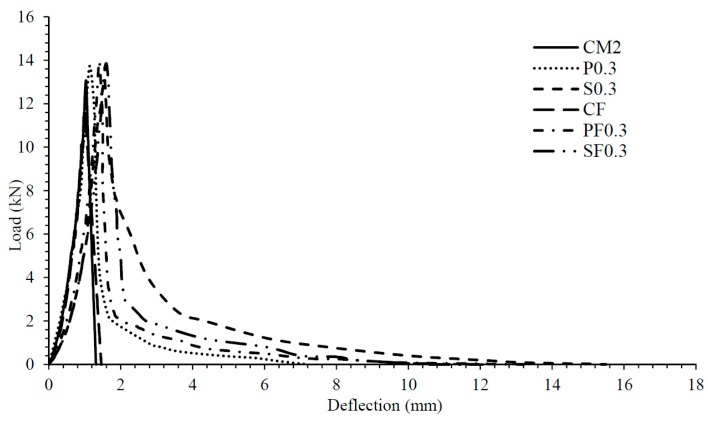
Load-deflection curve.

Figure 5 shows the results of other mechanical properties of concrete; the addition of PVA and CCS fibers also improved the split tensile strength and flexural strength of all concrete mixes. For 100% cement concrete; both PVA and CCS fibers caused a considerable increase in the ductility and the flexural toughness. An average increment of 77% and 265% was noticed, respectively. Similarly, in the case of fly ash concrete, PVA and CCS fibers increased the flexure toughness by 114% and 175%, respectively. Elastic modulus is one of the characteristics of concrete that influences the end zone behavior during the transfer of post-tension force. Addition of fibers PVA and CCS improved the elastic modulus of concrete. 100% cement concrete with PVA fiber showed the value of elastic modulus as 47 GPa, which is considerably high for enhancing the end zone performance. Figure 6 shows the load-deflection curves of all concrete mixes tested for flexural strength, which indicates that controlled mixes exhibited brittle behavior because the load was abruptly dropped after reaching the peak value. However, fiber reinforced concrete mixes (PVA & CCS) have experienced a large deflection after reaching the peak load, which may qualify such type of concrete as the ductile concrete. Therefore, based on the performance enhancement of concrete achieved with the addition of PVA and CCS fibers, it was anticipated that such concrete could improve the performance of the end zone of PT beams.

### 3.2. The Response of Beams at the Transfer of the Post-Tension Force

**Figure 7:**
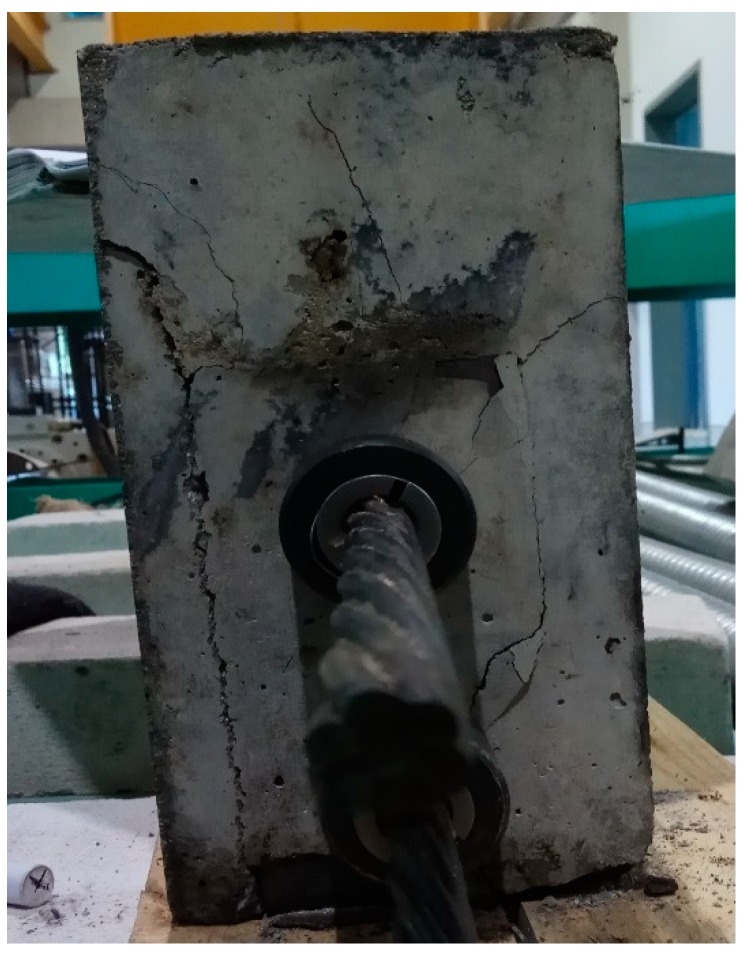
Cracks formation at transfer in fly ash based control beam, PSCFy-1.

A total of six beams were cast, at the age of seven days. A post-tensioning operation was performed for each of the beams using the pre-stressing bed located in the concrete laboratory at UTP. This is schematically drawn in Figure 3. Application of FRC using PVA and CCS fibers in the PT-Beams has shown some new knowledge and concepts. When the controlled beam was made of fly ash, PSCFy-1 was stressed to the full design force of 200 kN; the hairline cracks started to appear during the transfer of prestressing force, which became significant when the estimated magnitude of 200 kN PT-force was transferred. According to Breen et al. [5], vertical and diagonal cracks during pre-stress transfer stage occurred due to the spread of forces in the transverse direction and building-up of spalling stresses at the edges. This phenomenon, spalling of concrete at the bottom and formation of a full crack along the transverse dimension, is shown in Figure 7. Spalling stresses are generated because of large eccentricity and a large loading area in the transverse direction. Formation of such cracks causes loss of prestressing force. Therefore, flexural strength is reduced during service. The behavior of the PSCFY-1 beam was observed to be brittle as compared to the 100% OPC concrete.

Consequently, the fly ash concrete beam without fiber, PSCFY-2 was stressed until the hairline cracks appeared at the surface. The beam was stressed until 125 kN force, which was 37.5% lower than the designed pre-stress force. The fly ash concrete beam PSCFyPVA-0.3 containing 0.3% PVA fibers and 50% end zone reinforcement content was also stressed until hairline cracks appeared. It sustained 150 kN PT force, which indicates that the addition of PVA fiber enhanced the capacity of prestressing force by 20%. The other three beams were made of 100% OPC; one control beam and one beam each containing PVA and CCS fibers. All three beams were stressed until the full design pre-stress force (200 kN); in such beams, no hairline cracks appeared until the entire design load transferred. 

From the observations made on six beams during the transfer of prestressing force and the related discussion, it can be generalized that concrete beams containing fly ash can sustain 67% to 75% of the design post-tensioned force as compared to 100% cement concrete beams. Thomas [30] discussed the reason that at an early age, i.e., at the period of 7 days, usually fly ash is not reacted. It has been recognized that the pozzolanic activity starts after 28 days. Therefore, such beams are stressed at a lower force level than the OPC concrete. 

Structural Response of End-Anchorage Zone at applied load (service condition).

Structural response of anchorage end can be described as the variation in strain in the embedded steel stirrups and surface concrete. The following section discusses the anchorage end zone response of six beams made of fly ash and 100% cement concrete.

#### 3.2.1. End Zone Performance of Fly Ash Concrete Beams

**Figure 8:**
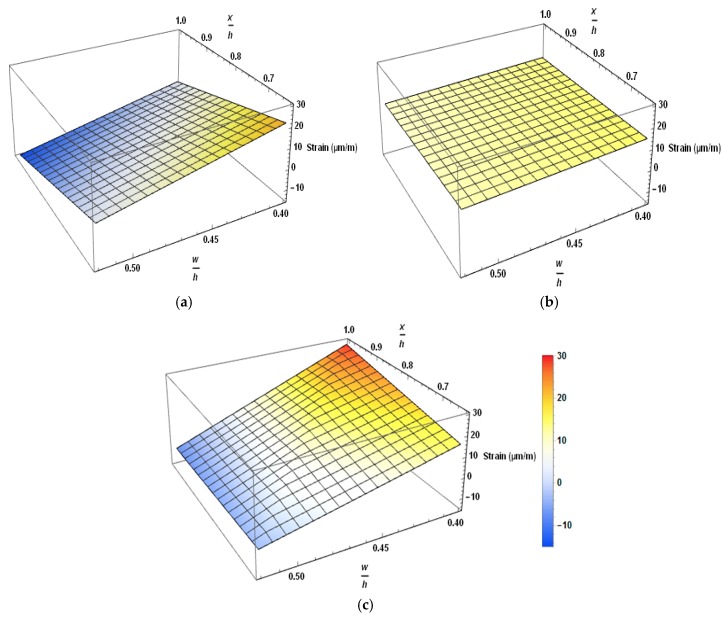
Strain variation in the anchorage end of fly ash concrete post-tensioned (PT) beams (**a**) PSCFy-1 (**b**) PSCFY-2 (**c**) PSCFY-PVA-0.3.

Figure 8 shows the strain variation of the local and general zone of fly ash concrete beams using three-dimensional representation. In this figure, strain values are plotted against (x/h) and (w/h) values, where (x/h) represents the normalized longitudinal dimension of the beam and (w/h) is the normalized length along the width. The letter w denotes the distance from the left edge of the member cross-section. The inner side of 3D graphs represents the strain variation in the steel stirrups, and the outer face represents the strain variation in the surface concrete. Dark blue and red colors represent the extreme tensile and compressive strains, respectively. Beam PSCFY-1 showed the perceived behavior; bursting tensile strain vanished at the end of the general zone and declined tensile phase was observed between local and general zones. Surface concrete showed an increase in compression strain (opposite to the bursting strain behavior). In the reduced design prestress (RDP) beam; the whole region showed small value and almost uniform tension, as shown in the Figure 8b. Peak tensile stresses occurred from the far end of the local zone to the last point of the general region, which may be due to very little change in strain values. Surface concrete was in tension even at the end of Anchorage. Reduction in the prestress force resulted in lower peak tensile strain and expanded the stress distribution area. The decrease in end zone reinforcement increases the tensile strain, as shown in Figure 8c. Stress distribution was still in the inclined tensile zone between local and general zones. Surface concrete exhibited the typical behavior of compression. PVA FRC beams managed to restrain the tensile strain even when the end zone reinforcement was reduced to half. Reduction in the end zone reinforcement increases the peak tensile stress value and expands the stress distribution length. 

**Figure 9:**
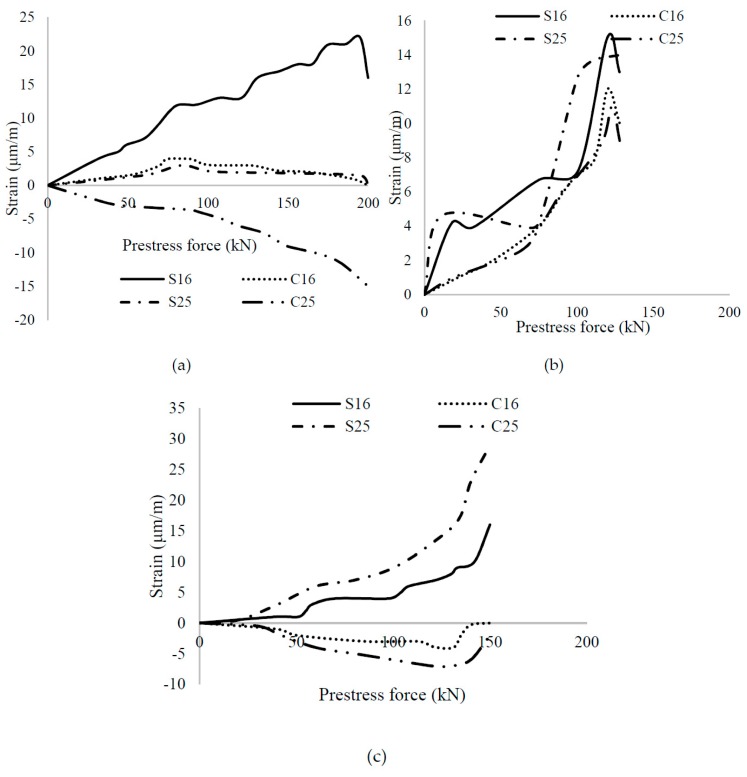
Strain variation with the change in the prestress force in fly ash concrete beams (**a**) PSCFy-1 (**b**) PSCFy-2 (**c**) PSCFyPVA-0.3.

Figure 9 describes the strain variation during the transfer of prestressing force; strain values were taken from different strain-gauge, as illustrated in Figure 2. The letter S is indicating the strain gauge on steel and the letter C represents the strain gauge on the surface concrete, whereas the number indicates the distance (cm) from the loading phase. As shown in the figure, the addition of fibers decreased the slope of the inclined graph in tension, which was due to an increase in ductility. While the controlled beam of fly ash shows a sudden change in the strain because of brittle behavior, the end drop in case of PSCFy-2 beam shows the growth of minor cracks or loss of prestressing force.

#### 3.2.2. End Zone Performance of 100% Ordinary Portland Cement (OPC) Beams

**Figure 10:**
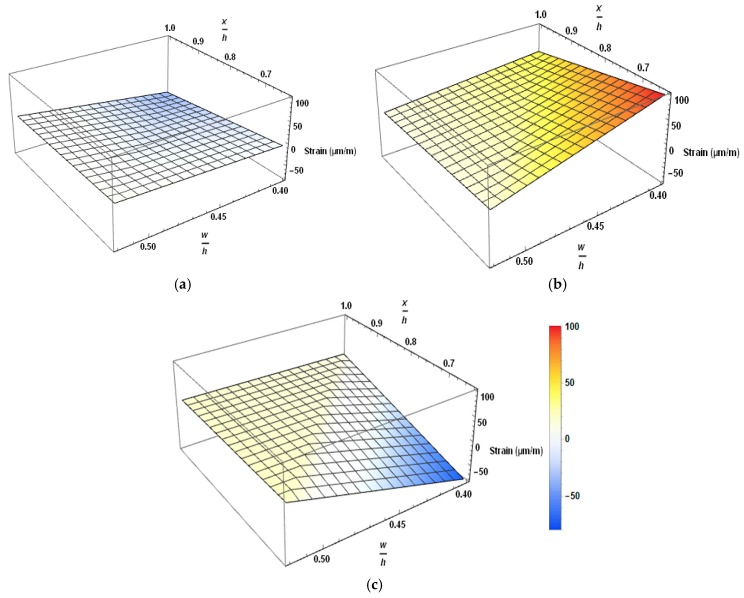
Strain variation in the anchorage end of 100% Ordinary Portland Cement (OPC) concrete beams (**a**) PSC (**b**) PSCPVA-0.3 (**c**) PSCS-0.3.

Figure 10 shows the maximum strain variation in the 100% OPC concrete beams. Such concrete gained enough strength at the age of 7 days. Hence, it could sustain the 100% design prestress force (200 kN). Control beam PSC showed minimal bursting strain that vanished at the end of the local zone, and compressive strains were developed from there onwards. Similarly, surface concrete also showed a small tensile strain. Usually, concrete acts as a layer, i.e., little compression at the center causes low tension at the edges, like Saint Venant’s principle as discussed by Toupin [31]. In the case of PSC-PVA beam, a decline of tensile zone occurred between local and general zone. It means that the peak tensile point happened within the anchorage zone. Despite providing half end zone reinforcement in the beam, PVA responded well to detain the expansion of bursting stresses far away from the anchorage zone because of the high elastic modulus, whereas steel FRC beam showed compressive strain dominant behavior, which was opposite to the PVC FRC beam. At the initial stage, stress distribution was observed between local zone and general zone. Both compression and inclined tensile zones occurred between local zone and general zone. Peak tensile stress point located beyond the anchorage end. For maintaining equilibrium, surface concrete displayed high tensile stresses [8]. Strain in the surface concrete remained in tension alike PSC and PSC-PVA beams. However, this time strain was high and almost constant with the change in distance. 

**Figure 11:**
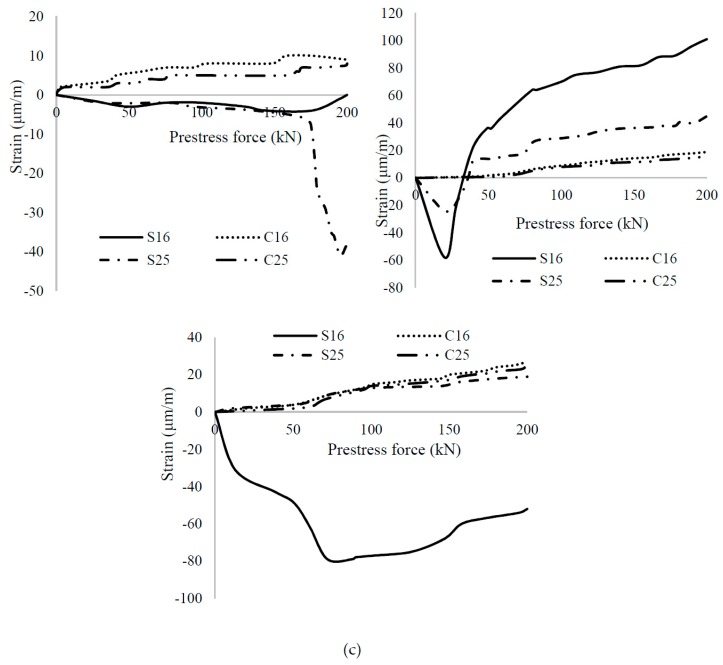
Strain variation with change in prestress force in 100% Ordinary Portland Cement (OPC) concrete beams (**a**) PSC; (**b**) PSCPVA-0.3; (**c**) PSCS-0.3.

Figure 11 shows the strain variation following the change in the magnitude of the prestressing force. In the control beam, an abruptly increased strain was observed at the end of the general zone at the transfer of 170 kN of prestressing force, which indicates the brittle behavior. PVA FRC showed initial compression strain in steel stirrups but later shifted to tension strain, as shown in Figure 11b. Tensile strain reduced by 56% at the end of the general zone, compared to the strain at the end of the local zone in steel stirrups. Uniform and the small tensile strain was observed in the surface concrete, where a 16% reduction in strain was found. Significant performance of PVA FRC was because of the high elastic modulus. 

Overall, it is observed that the addition of fly ash as a partial substitute of cement enhances tensile stresses in the end region that become a cause of cracks. RDP reduced the peak tensile value but increased the area under the influence of tensile stresses. Reduction in the amount of end zone reinforcement encourages bearing compression stresses, tensile bursting stresses, and transmission length. Addition of PVA fibers managed the effects of modification of end zone reinforcement. In contrast, the anchorage zone of CCS FRC trapped in the compression stresses and expanded the stress distribution area far beyond the anchorage zone. Anchorage zone behavior of post-tension beams can be linked with the elastic modulus of the specimen. 

### 3.3. Strain-Energy and Change in Maximum Strain

**Figure 12:**
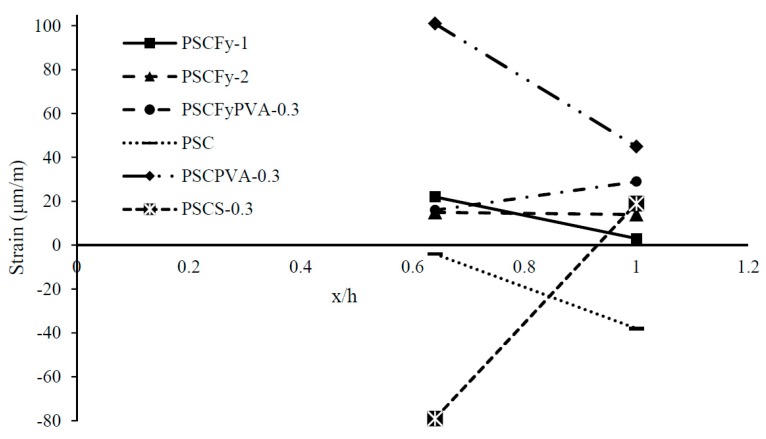
Change in maximum strain in shear links at different distance (x/h).

**Figure 13:**
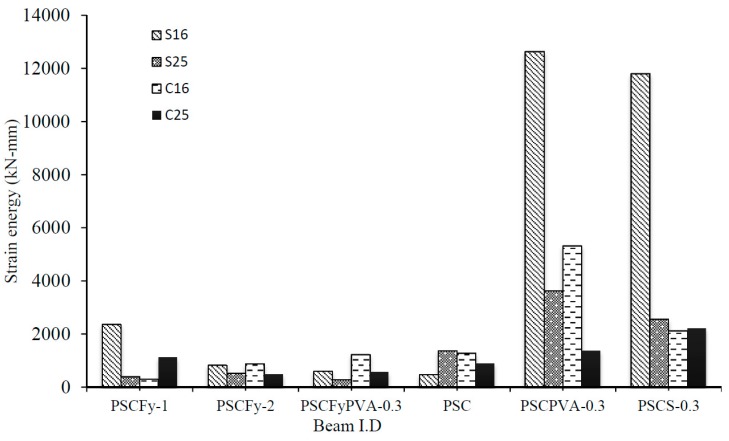
Strain energy distribution in the end zone.

Strain energy shows the energy needed for failure or to bring some strain. It is estimated as the area under the curve. Usually, high energy dissipated material is required in critical structures to resist the high impact and dynamic load. Therefore, fiber is used to increase the ductility and energy absorption capacity of concrete [32]. The strain energy distribution is also critical to estimate the ductility of prestressed concrete structure [33]. Figure 12 shows the strain energy measured using the data of every strain gauge installed on the beams. Figure 13 demonstrates that the addition of fiber in 100% OPC mixes increased the ductility of the end region by many times. Total strain energy values of fly ash concrete beams; PSCFy-2 and PSCFyPVA-0.3 found closer to the toughness value calculated when the first crack appeared. 100% OPC fiber reinforced concrete beams showed much higher energy dissipation; such beams were stressed to full design force and were not cracked. In Figure 13, S16 represents the strain energy in the first prism, which is the highest critical zone in the anchorage area. Control beam (PSC) showed the least value of the strain energy in the first prism, whereas the beams made of FRC mixes showed a significantly high amount of strain energy. PVA FRC and copper coated micro steel FRC amplified the strain energy in the local zone by 26 and 24 times, respectively. PVA fiber exhibited better ductility than steel fiber. 

Figure 12 demonstrates the change in maximum internal strain along the longitudinal direction. The graph exhibits a very disturbed sequence because of the application of high prestress force on a small cross-section. Fibre reinforced concrete lines also display the effect of reduction in end zone reinforcement and fiber response on stress distribution. Mixes with fly ash present very less strain because of rigid behavior in the early stage of the maturity process. PSC shows compression and less strain. PVA FRC and steel FRC exhibit the ductile behavior by increasing the slope of strain line. Both the fiber displays very sharp inclines mean stress dissipate with distance effectively. Tensile strain reduced by 55% in PVA fiber reinforced concrete with moving 90 mm distance. However, steel fiber reinforced concrete is still in the process of the first phase of stress distribution, i.e., from compression to tension. Literature shows that high eccentricity and loading area increases the expansion of stresses to longer distance. In the case of fiber reinforced concrete, the strain variation is high with the length. Reduction of end zone reinforcement expands the stress distribution beyond the anchorage zone.

**Figure 14:**
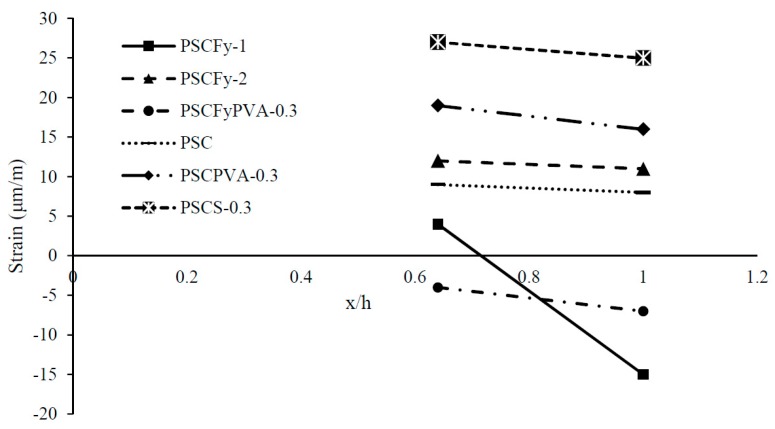
Change in maximum strain on the concrete surface at different distance (x/h).

Figure 14 highlighted the more precise view of the variation in strain with distance. Axial prestress force does not impact the surface concrete directly but transfer stresses according to Saint Venant’s principle. PVA FRC drops the strain line into compression zone. PVA and steel fiber reinforced concrete mixes increased the ductility of brittle HSC by raising the strain line with a slight increase in slope. Overall, it is observed that control beams and fly ash concrete beams exhibit less energy dissipation and less strain variation with distance, however, fiber reinforced concrete without fly ash significantly increased the ductility of the end zone.

## 4. Conclusions

Form the results and discussion on the experimental investigation and the analysis performed, the following conclusion can be drawn: 

Both types of fibers (0.3% by the volume fraction of PVA and CCS) in the fiber reinforced concrete used in the study have enhanced the ductility and mechanical properties of concrete. PVA fibers caused much higher effects on the elastic modulus of concrete, particularly in 100% OPC concrete, while PVA fibers significantly increased the elastic modulus to 47 GPa. 

Pozzolanic reactivity of fly ash usually starts after 28 days of curing. Since PT transfer was made at the age of 7 days, at that age fly ash was found unreacted in concrete. Therefore, the design prestressing force for fly ash concrete beams was reduced to about 38%.

From the analysis of the end zone strain of all PT beams, it is observed that the inclusion of fly ash increased the tensile stresses, which caused cracks to appear. Therefore, decreasing the design prestress level reduced the induction of tensile stresses, but it raised the tensile stress distribution area. Reduction in the end zone reinforcement caused an increase in compression stresses, tensile stresses, and stress distribution area. PVA FRC beams managed to withstand the high intensity of stresses and restrained the drastic changes to occur in the end zone behavior. On the other hand, copper coated micro steel FRC even did not handle well the compression stresses and allowed to exceed beyond the local zone.

PVA and copper coated steel fibers in FRC resulted in the reduction of the end zone reinforcement and avoided the congestion that caused a smooth transmission of prestressing force. 

This research can be extended by involving the loading area, the eccentricity of the tendon, and percentage of the end zone reinforcement as a variable and generate a guideline for the FRC PT-beam on both experimental and analytical levels.

## Figures and Tables

**Table 1:** Physical and Mechanical Properties of fibers.

Properties	Copper Coated Steel (CCS) Fiber	PVA Fiber
Length (mm)	20	18
Diameter (μm)	200	200
Aspect Ratio (*l/d*)	100	90
Density (g/cm^3^)	7.85	1.3
Tensile strength (MPa)	2300	1000

**Table 2:** Details of concrete mix proportions.

Mix ID	Fiber Dosage (by Volume) (%)		Cement (kg/m^3^)	Fly Ash (kg/m^3^)	Coarse Aggregate (kg/m^3^)	Fine Aggregate (kg/m^3^)	w/c Ratio	SP (%)
	PVA	CCS						
CM	0	0	425	0	1100	705	0.4	0.35
P0.3	0.3	0	425	0	1100	705	0.4	0.4
S0.3	0	0.3	425	0	1100	705	0.4	0.4
CF	0	0	340	85	1100	705	0.4	0.25
PF0.3	0.3	0	340	85	1100	705	0.4	0.3
SF0.3	0	0.3	340	85	1100	705	0.4	0.5

SP—superplasticizer; w/c—water to cementitious binder ratio.

**Table 3:** Post-tensioned prestressed beams details.

Beam ID	Mix Type	Prestressing Force (kN)	End-Zone Reinforcement, (%)	Remarks
PSCFy-1	CF	200	100	Cracked at the end, because fly ash concrete gain low strength at an early age.
PSCFy-2	CF	125	100	Hairline crack on the surface, for fly ash concrete, this load is considered ultimate Pre-Stress Force
PSCFyPVA-0.3	PF0.3	150	50	Hairline crack on surface
PSC	CM	200	100	Successful
PSCPVA-0.3	P0.3	200	50	Successful
PSCS-0.3	S0.3	200	50	Successful

**Table 4:** Experimental Program of the research.

Test	Specimen Age at Testing, (Days)	No. of Samples (for each mix)	Size of Mold (mm)
Compression strength, (MPa)	3	3	100 × 100 × 100
	7	3	100 × 100 × 100
	28	3	100 × 100 × 100
	56	3	100 × 100 × 100
Split tensile strength, (MPa)	28	3	100 diameter × 200 height
Flexure Strength, (MPa)	28	3	100 × 100 × 500
Elastic Modulus, (GPa)	28	3	100 diameter × 200 height
Post-tensioning	7	1	130 × 250 × 2000

**Table 5:** Cube Compressive Strength Test Results.

Mix Type	3 Days		7 Days		28 Days		56 Days	
	f_cu_	SD	f_cu_	SD	f_cu_	SD	f_cu_	SD
CM	54.1	2.2	60.5	1.4	66.8	2.1	70.9	2.5
P0.3	54.3	1.6	60.5	0.5	69.0	2.9	73.9	1.6
S0.3	58.9	2.7	65.8	2.5	72.6	1.1	78.3	0.9
CF	42.9	0.9	50.1	2.3	66.0	2.0	72.0	1.8
PF0.3	35.4	1.0	55.6	1.8	64.0	1.9	74.5	2.0
SF0.3	46.2	1.2	55.4	1.4	54.4	10.6	83.6	1.7

f_cu_—the cube compressive strength; SD—the standard deviation of the THREE samples result.
